# Pulmonarom^®^ promotes TLR-related and interferon-related antiviral responses during influenza A (H1N1 and H3N2) infection in human monocyte-derived dendritic cells

**DOI:** 10.3389/fcimb.2026.1891757

**Published:** 2026-08-18

**Authors:** Sandra Georgina Solano-Gálvez, Juan A. Hernández-Aceves, Alberto García Lozano, Arturo A. Wilkins-Rodríguez, Diego Alexander Rojas Ortega, Antonio Ibarra, Exsal Manuel Albores Méndez, Edda Sciutto, Laila Gutiérrez-Kobeh, Horacio Merchant Larios, Carlos Cabello-Gutiérrez, Gladis Fragoso, Rosalino Vázquez-López

**Affiliations:** 1 Unidad de Investigación Universidad Nacional Autónoma de México – Instituto Nacional de Cardiología (UNAM-INC), División de Investigación, Facultad de Medicina, Ciudad de México, Mexico; 2 Departamento de Inmunología, Instituto de Investigaciones Biomédicas, Universidad Nacional Autónoma de México, Ciudad de México, Mexico; 3 Laboratorio de Inmunobiología, Departamento de Biología, Facultad de Química, Universidad Nacional Autónoma de México, Ciudad de México, Mexico; 4 Centro de Investigación en Ciencias de la Salud (CICSA) Facultad de Ciencias de la Salud Universidad Anáhuac México Norte, Huixquilucan, Mexico; 5 Escuela Militar de Graduados en Sanidad Universidad del Ejército y Fuerza Aérea Secretaría de la Defensa Nacional México, Ciudad de México, Mexico; 6 Laboratorio de Biología Celular y Fisiología Instituto de Investigaciones Biomédicas, Universidad Nacional Autónoma de México, Ciudad de México, Mexico; 7 Departamento de Investigación en Virología y Micología, Instituto Nacional de Enfermedades Respiratorias (INER), Ciudad de México, Mexico

**Keywords:** antiviral, dendritic cells, immunomodulators, influenza virus (H1N1 and H3N2), interferon, postbiotic, Pulmonarom^®^, TLR

## Abstract

**Background:**

Bacterial lysates have been associated with improved host defense against respiratory infections; however, their mechanisms of action remain incompletely understood. Based on our previous *in vitro* work showing that Pulmonarom^®^ activates human monocyte-derived dendritic cells (moDCs) and induces TLR- and cytokine-related responses consistent with innate immune activation, we evaluated its immunomodulatory effect during influenza A virus infection using two *in vitro* conditions: pre-exposure and post-infection treatment.

**Materials and methods:**

Human monocytes obtained from buffy coats were differentiated into moDCs. Lyophilized Pulmonarom^®^ was quantified as total protein and evaluated at 0.01, 0.1, or 0.5 µg/mL in 24-well cultures containing 1 × 10^6^ moDCs in 1 mL medium per well. In the pre-exposure condition, moDCs were incubated with Pulmonarom^®^ for 1 h before infection with influenza A/H1N1 or A/H3N2 virus. In the post-infection treatment condition, Pulmonarom^®^ was added after the 1-h infection period. TLRs and cytokine expression were evaluated by flow cytometry. Changes in hemagglutinating activity were evaluated by hemagglutination inhibition assay and virus infectivity was evaluated with a MDCK plaque cell assay.

**Results:**

Under both pre-exposure and post-infection treatment conditions, Pulmonarom^®^ enhanced MHC class II expression and improved cell viability following infection with either A/H1N1 or A/H3N2 virus, consistent with moDC activation. In hemagglutination inhibition assays, Pulmonarom^®^ was associated with reduced hemagglutinating activity in culture supernatants. Similarly, plaque infectivity assays demonstrated that Pulmonarom^®^, administered either before or after infection, decreased the number of lysed cells within plaques, with a more pronounced effect observed under post-infection treatment. These immunomodulatory changes were accompanied by increased cytokine secretion and the induction of type I interferon-related responses.

**Conclusions:**

Pulmonarom^®^ modulated human moDC responses during *in vitro* infection with influenza A/H1N1 and A/H3N2 virus. These findings extend our previous observations on Pulmonarom^®^-induced innate immune activation in human dendritic cells and support further analysis of this bacterial lysate as a host-directed immunomodulatory strategy. Future studies should be evaluated to determine infectious viral titers, TLRs dependency, dendritic-cell maturation markers, and downstream T-cell responses; using plaque assay with sensible cells, receptor-blocking approaches, expanded maturation and characterization panels, and functional co-culture assays.

## 1 Introduction

Seasonal influenza remains a major public health problem worldwide because it causes recurrent epidemics associated with substantial morbidity and mortality (The Economic Risks and Impacts of Epidemics - IMF F&D Magazine - June 2018 | Volume 55 | Number 2; Naghavi et al., 2024; Caballero et al., 2025). Circulating influenza A viruses continue to change, and A(H3N2) frequently accounts for a large proportion of isolates and is often associated with more severe disease, particularly in older adults (The Economic Risks and Impacts of Epidemics - IMF F&D Magazine - June 2018 | Volume 55 | Number 2; Naghavi et al., 2024; Caballero et al., 2025). Influenza A(H1N1) pdm09, the swine-origin strain responsible for the 2009 pandemic, also continues to circulate seasonally. Despite the availability of vaccines, antigenic drift and the risk of new variants support the need for additional strategies that reinforce early host defenses (Seasonal Influenza - Global Situation).

Most acute respiratory infections are viral; therefore, antibiotics are not indicated unless bacterial co-infection is suspected or confirmed. Nevertheless, inappropriate antibiotic prescription for acute respiratory tract infections remains common and contributes to antimicrobial resistance (AMR), a major global health threat (O’Connor et al., 2018) In this context, the continued evolution of influenza viruses and the increasing pressure from AMR highlight the need for preventive and host-directed approaches that can reduce infection risk and disease severity.

Early innate immune recognition and cytokine production strongly influence the course of influenza virus infection. Dendritic cells (DCs) play a central role in antiviral immunity by sensing pathogens, producing inflammatory mediators and interferons, and initiating adaptive immune responses (Areschoug and Gordon, 2008; Dowling and Mansell, 2016). DCs activation is commonly evaluated through increased expression of major histocompatibility complex class II (MHC-II) molecules and changes in pattern-recognition receptors, particularly Toll-like receptors (TLRs), which recognize microbial and viral components and guide cytokine production (Areschoug and Gordon, 2008; Dowling and Mansell, 2016). Several immunomodulatory strategies have therefore focused on strengthening innate antiviral responses through TLR stimulation and DC activation, including synthetic agonists such as imiquimod (TLR7) and monophosphoryl lipid A (TLR4), as well as bacterial-based approaches such as BCG and defined bacterial components (Mata-Haro et al., 2007; Dowling and Mansell, 2016; Moulson and Av-Gay, 2020; Ng et al., 2020; Tepale-Segura et al., 2024).

Bacterial lysates have long been used clinically to prevent recurrent infections, particularly in the respiratory tract (Morandi et al., 2011; Coviello et al., 2014). Although beneficial effects have been reported in different clinical settings, their mechanisms of action remain incompletely understood. One proposed mechanism is that bacterial lysates provide multiple ligands capable of engaging pattern-recognition receptors and thereby activating or functionally programming immune cells, including DCs (Zelle-Rieser et al., 2001; Areschoug and Gordon, 2008). This host-directed mechanism may be relevant for respiratory viruses such as influenza, in which early activation of innate immune pathways and interferon responses can influence viral replication and subsequent inflammation (Beirag et al., 2025).

Pulmonarom^®^ is a commercially available multicomponent bacterial lysate used for the prevention and adjunctive treatment of respiratory infections. According to the product information, each 3 mL ampoule contains lysates derived from *Haemophilus influenzae*, *Staphylococcus aureus*, *Moraxella catarrhalis*, *Klebsiella pneumoniae*, *Streptococcus pneumoniae*, *Streptococcus pyogenes*, *Streptococcus agalactiae*, *Streptococcus dysgalactiae*, and *Streptococcus anginosus*, each corresponding to 6 × 10^8^ colony-forming-unit equivalents (Plm - Mexico (2026) Pulmonarom - Solución; Roth et al., 2021). Because Pulmonarom^®^ is a whole bacterial lysate rather than a purified single-molecule drug, its biological activity likely reflects the combined action of multiple microbial-associated molecular patterns, including peptidoglycan, lipoteichoic-acid-associated structures, bacterial lipoproteins, nucleic acids, and Gram-negative envelope components such as lipooligosaccharide/lipopolysaccharide-related molecules. Therefore, experimental dosing needs to be normalized by total protein content.

Previous studies support the immunomodulatory activity of Pulmonarom^®^ in human myeloid cells. *In vitro*, Pulmonarom^®^ activates human dendritic cells and induces features consistent with innate immune responsiveness (Hernández-Aceves et al., 2025). In monocyte/macrophage models of mycobacterial infection, Pulmonarom^®^-related studies have also shown modulation of innate defense pathways, including BPI induction and autophagy-associated antimicrobial responses (García-González et al., 2025; Romero-Rodríguez et al., 2025). These observations are relevant because bacterial lysates may display product-specific antiviral properties, emphasizing the importance of defining both the composition of the lysate and the experimental preparation used in each study (Roth et al., 2021).

Based on this background, we investigated whether Pulmonarom^®^ modifies antiviral sensing and cytokine responses during influenza A virus infection in human moDCs. Although Pulmonarom^®^ may also interact with primary monocytes and macrophages *in vivo*, freshly isolated primary human monocytes were not independently evaluated in the present influenza virus experiments before differentiation into moDCs. Therefore, direct analysis of monocyte viability, activation markers, and inflammatory cytokine production remains an important objective for future studies.

## 2 Materials and methods

### 2.1 Generation of monocyte-derived dendritic cells

Monocyte-derived dendritic cells (moDCs) were generated from CD14+ monocytes as previously described (Romani et al., 1994; Sallusto and Lanzavecchia, 1994). Peripheral blood monocytes were isolated from anonymized buffy coats obtained from healthy donors, kindly provided by the Blood Bank of the National Institute of Cardiology ‘Ignacio Chávez.’ All procedures were conducted in accordance with the Declaration of Helsinki. Written informed consent was obtained from all donors. The study protocol was approved by the Comité de Ética de la Investigación, División de Investigación, Facultad de Medicina, Universidad Nacional Autónoma de México (UNAM), protocol FM/DI/114/2019, approved on 4 August 2020.

Peripheral blood mononuclear cells (PBMCs) were isolated by density gradient centrifugation with Histopaque-1077 (Sigma-Aldrich; Merck KGaA, Darmstadt, Germany). Briefly, buffy coats were diluted 1:2 with Dulbecco’s phosphate-buffered saline (D-PBS) and centrifuged at 400 × g for 45 min at 24 °C. The mononuclear cell layer was collected and washed three times with D-PBS (300 × g, 10 min, 4 °C). Cells were resuspended in blocking buffer (D-PBS containing 2 mM EDTA and 0.5% BSA, cell culture grade) and incubated at 4 °C for 15 min.

For monocyte isolation, cells were incubated with anti-CD14 magnetic microbeads (Miltenyi Biotec, Bergisch Gladbach, Germany) for 20 min at 4 °C, washed with D-PBS, and separated using LS magnetic columns (Miltenyi Biotec) according to the manufacturer’s instructions. The columns were washed three times with 3 mL of D-PBS to remove CD14^-^ cells, then detached from the magnet to recover CD14^+^ monocytes using a plunger and D-PBS.

Purified monocytes were counted and seeded at a density of 1 × 10^6^ cells/mL in RPMI-1640 medium with stable glutamine (Biowest, Riverside, MO, USA), supplemented with 10% FBS, 100 U/mL penicillin, 100 µg/mL streptomycin, and 50 µM 2-mercaptoethanol (pH 7.2), hereafter referred to as R-10 medium. Cells were cultured in 24-well tissue culture-treated plates.

On the following day, 50% of the medium in each well was replaced with fresh R-10 medium supplemented with 500 U/mL granulocyte-macrophage colony-stimulating factors (GM-CSF) and 1000 U/mL interleukin-4 (IL-4) (BD Biosciences, Franklin Lakes, NJ, USA) to induce differentiation into immature moDCs. This medium replacement was repeated on days 2 and 4 of culture. Immature moDCs were harvested on days 5–6 and used for the subsequent assays.

### 2.2 Pulmonarom^®^ Lyophilization and protein quantification

Commercial bacterial lysate Pulmonarom^®^ was purchased from a pharmacy as 3 mL glass ampoules. The product contains bacterial lysates derived from *Haemophilus influenzae, Staphylococcus aureus, Moraxella catarrhalis, Klebsiella pneumoniae, Streptococcus pneumoniae, Streptococcus pyogenes, Streptococcus agalactiae, Streptococcus dysgalactiae*, and *Streptococcus anginosus*, each corresponding to 6 × 10^8^ colony-forming-unit equivalents per ampoule (Plm - Mexico (2026) Pulmonarom - Solución; Roth et al., 2021). Because the product is a multicomponent lysate, no single purified active pharmaceutical ingredient can be assigned. Pulmonarom^®^ was therefore handled as the complete bacterial lysate, and the experimental dose was normalized by total protein content. The 3 mL content of each ampoule was lyophilized (-120 °C, Maxi Dry Lyo). Protein concentration was quantified using the Bradford method (Bradford, 1976) The lyophilized powder was stored until use and reconstituted to the desired concentration before experiments. Endotoxin/LPS content was not quantified in these experiments. Therefore, the contribution of endotoxin-containing Gram-negative components cannot be separated from other lysate-derived MAMPs/PAMPs under the current conditions.

### 2.3 Pulmonarom^®^ stimulation of moDCs and flow cytometric characterization

Monocyte-derived dendritic cells were seeded in 24-well culture plates at 1 × 10^6^ cells in 1 mL medium per well and treated with Pulmonarom^®^ at final total-protein concentrations of 0.01, 0.1, or 0.5 µg/mL (equivalent to 0.01, 0.1, or 0.5 µg protein per 10^6^ cells per well). Two *in vitro* conditions were used: (i) pre-exposure, in which moDCs were incubated with Pulmonarom^®^ for 1 h before influenza virus infection; and (ii) post-infection treatment, in which Pulmonarom^®^ was added after the 1-h infection period, as described below. After incubation, culture supernatants were collected and stored at -80 °C until analysis, and cells were processed for flow cytometry.

For moDC characterization, cells were recovered, cooled to 4 °C for 30 min, and washed with cold phosphate-buffered saline (PBS; 137 mM NaCl, 2.7 mM KCl, 10 mM Na2HPO4, 1.8 mM KH2PO4). Cell number and viability were determined by trypan blue exclusion; only samples with viability >85% were included. Cells were resuspended in FACS buffer (PBS supplemented with 1% bovine serum albumin [BSA]) and adjusted to 5 × 10^5^ cells per tube. Non-viable cells were excluded during flow-cytometry analysis using the Zombie Aqua Fixable Viability Kit (BioLegend, Cat. 423102). After washing, surface staining was performed by incubating cells with antibodies in FACS buffer for 30 min at 4 °C, followed by PBS washes. Surface antibodies included anti-CD11c-PECy7 (BioLegend, Cat. 337216), anti-HLA-DR-AF700 (BioLegend, Cat. 307626), anti-TLR2-AF647 (BioLegend, Cat. 309714), anti-TLR4-BV421 (BioLegend, Cat. 312811), and anti-TLR6-PE (BioLegend, Cat. 334708). Isotype controls included PE Mouse IgG2a κ (BioLegend, Cat. 400211) and APC Mouse IgG1 κ (BioLegend, Cat. 400119). Trypan blue exclusion was used only as a sample-quality criterion before staining.

After surface staining, cells were fixed with Fixation Buffer (BioLegend, Cat. 420801) for 20 min at room temperature, then washed with 1× Wash Buffer (BioLegend, Cat. 421002) and centrifuged twice at 1500 rpm for 5 min. Cells were incubated overnight (~12 h) in 1× Wash Buffer containing intracellular antibodies: anti-TLR3-BV711 (BioLegend, Cat. 309714), anti-TLR7-FITC (BioLegend, Cat. 376908), anti-TLR8-PerCP-Cy5.5 (BioLegend, Cat. 395512), and anti-TLR9-APC-Fire™ 810 (BioLegend, Cat. 394812). Finally, cells were washed with 1×Wash Buffer and acquired on a flow cytometer for analysis.

Samples were acquired on the Attune NxT flow cytometer (Thermo Fisher Scientific), which enables simultaneous detection of up to 13 fluorescence parameters. Data files were analyzed using FlowJo version 10.8 (BD Biosciences). The flow-cytometry panel in this study focused on viability, CD11c, HLA-DR/MHC-II, and TLRs expression.

### 2.4 Preparation of viral inoculum

Influenza A H1N1 and H3N2 viruses were cultured in MDCK (NBL-2) cells (ATCC^®^ CCL-34™) in Minimum Essential Medium (MEM) (Alpha Medium, 1X, with Earle’s salts, without ribonucleosides, deoxyribonucleosides, and L-glutamine, ATCC Catalog No. 30-2605), supplemented with 10% fetal bovine serum (FBS) (ATCC^®^ 30-2020), at 37 °C and 5% CO2.

Two influenza A virus subtypes were used in this study. The A/H1N1 virus was isolated from a clinical sample collected during the 2009 pandemic and characterized at the Department of Virology and Mycology Research, Instituto Nacional de Enfermedades Respiratorias (INER). The A/H3N2 virus was the ATCC^®^ strain VR-1680™. Use of the A/H1N1 clinical isolate was restricted to anonymized, previously characterized viral material, and experiments were performed under institutional biosafety procedures for handling human influenza A virus in cell culture. No patient identifiers or clinical data were used. Viruses were propagated in 25 cm² culture flasks containing MEM until the characteristic influenza virus-induced cytopathic effect was observed in the cell monolayer. Culture supernatants were collected, aliquoted, and stored at -70 °C until use. One aliquot of each viral stock was used to determine hemagglutination titers.

### 2.5 Influenza infection of moDCs

moDCs were infected with both influenza A virus subtypes (H1N1 and H3N2) at MOI = 1.0 when confluence was approximately 90%, based on previously described protocols. After a 1-h infection period at 37 °C in 5% CO2, the viral inoculum was removed, and cells were washed once with a salt solution supplemented with 20 mM HEPES, pH 7.4. Cells were suspended in serum-free growth medium and incubated at 37 °C with 5% CO2 for 24 h. The present study therefore evaluated a single 24 h post-infection time point. H1N1 and H3N2 experiments were performed at the same nominal MOI and analyzed at the same 24 h post-infection time point.

### 2.6 Cytokine production

Cytokines were quantified in cell supernatants after 24 h of stimulation with Pulmonarom^®^ using the Human Anti-Virus Response Panel 1 kit (BioLegend, Cat. 741270, San Diego, CA, USA) according to the manufacturer’s instructions. Briefly, supernatants were incubated with capture beads for 2 h and then washed with the provided wash buffer. Biotinylated detection antibodies were added and incubated for 1 h at room temperature with constant shaking (330 rpm on an orbital shaker). Without washing, streptavidin was added and incubated for an additional 30 min. Finally, the beads were washed and prepared for flow-cytometry analysis. The limit of quantification (LOQ) for each cytokine, as determined during kit calibration, was as follows: IP-10: 65.2 pg/mL; IL-1β: 0.89 pg/mL; TNF-α: 104.06 pg/mL; IL-6: 33.03 pg/mL; IL-10: 76.32 pg/mL; IFN-γ: 68.64 pg/mL; IL-12p70: 27.7 pg/mL; IL-8: 102.25 pg/mL; IFN-λ1: 249.4 pg/mL; IFN-λ2/3: 804.6 pg/mL; GM-CSF: 351.1 pg/mL; and IFN-β: 514.7 pg/mL.

### 2.7 Inhibition of the hemagglutination assay

Cell-culture supernatants were diluted 1:4 with PBS and then subjected to two-fold serial dilutions across the plate. In parallel, hemagglutinin particles (pHA) were adjusted to 4 units (1 pHA unit corresponds to the minimum concentration at which agglutination was observed). In U-bottom plates (VWR, Cat. 82050-622), 25 µL of PBS was first added, followed by 25 µL of diluted supernatant in the first row. Serial two-fold dilutions were then performed. For the positive virus control, 25 µL of diluted pHA was added; for the negative control, only 25 µL of PBS was added. The plate was gently mixed, and 50 µL of 10% guinea pig erythrocytes (Rockland, Cat. R402-0050) were added. After gentle mixing, the plate was incubated at room temperature for 1 h. The lowest dilution showing no agglutination was considered the hemagglutination inhibition titer. This assay provides an indirect estimate of hemagglutinating activity and cannot distinguish infectious virions from noninfectious or defective particles.

### 2.8 Viral plaque assay

The antiviral activity of Pulmonarom^®^ against influenza A/H1N1 and A/H3N2 viruses was evaluated by quantifying infectious viral particles in the supernatants of infected moDCs using a plaque assay. Viral titers were determined in Madin–Darby canine kidney (MDCK; NBL-2, ATCC^®^ CCL-34™) cells following a previously described method for influenza virus quantification, with minor modifications. Briefly, confluent MDCK monolayers were inoculated with serial dilutions of moDC supernatants (pre- and post-treated with Pulmonarom^®^ at 0.01, 0.1, and 5 μg) and maintained under semisolid overlay conditions to allow the formation of localized plaques arising from individual infectious virions. After the incubation period, cell monolayers were fixed, stained, and plaques were visually enumerated. Viral titers were calculated based on the number of plaques obtained at the appropriate dilution and expressed as plaque-forming units per milliliter (PFU/mL). All titrations were performed in independent experiments to ensure reproducibility of the results.

### 2.9 Transmission electron microscopy

moDCs were fixed with Karnovsky fixative (2.5% glutaraldehyde/2% formaldehyde) and washed with 0.1 M cacodylate buffer. Post-fixation was performed with osmium tetroxide (OsO4) for 2 h, followed by contrast staining with 1% uranyl acetate for 1 h and dehydration through increasing alcohol concentrations. Two changes of propylene oxide were performed for 30 min each. Samples were pre-embedded in propylene oxide/Epon resin (2:1 TN, 1:1 TN, and pure TN), and final inclusion was carried out at 60 °C. Semi-thin sections of 1 µm were prepared and stained with toluidine blue. Thin sections (60–70 nm) were contrasted with uranyl acetate and lead citrate and examined by transmission electron microscopy using a JEM-100C electron microscope (Jeol Ltd., Tokyo, Japan).

### 2.10 Statistical analysis

Data distribution was assessed with the Shapiro-Wilk test. Normally distributed variables were analyzed with parametric tests, whereas non-normally distributed variables were analyzed with non-parametric tests. For parametric analyses, group comparisons were performed with one-way ANOVA followed by Tukey’s *post hoc* test. Non-parametric data were analyzed with the Kruskal-Wallis test. Results are presented as mean ± standard deviation (SD), and statistical significance was set at p < 0.05.

## 3 Results

### 3.1 Pulmonarom^®^ increases MHC-II expression and supports moDC activation during influenza virus infection *in vitro*

**Figure 1:**
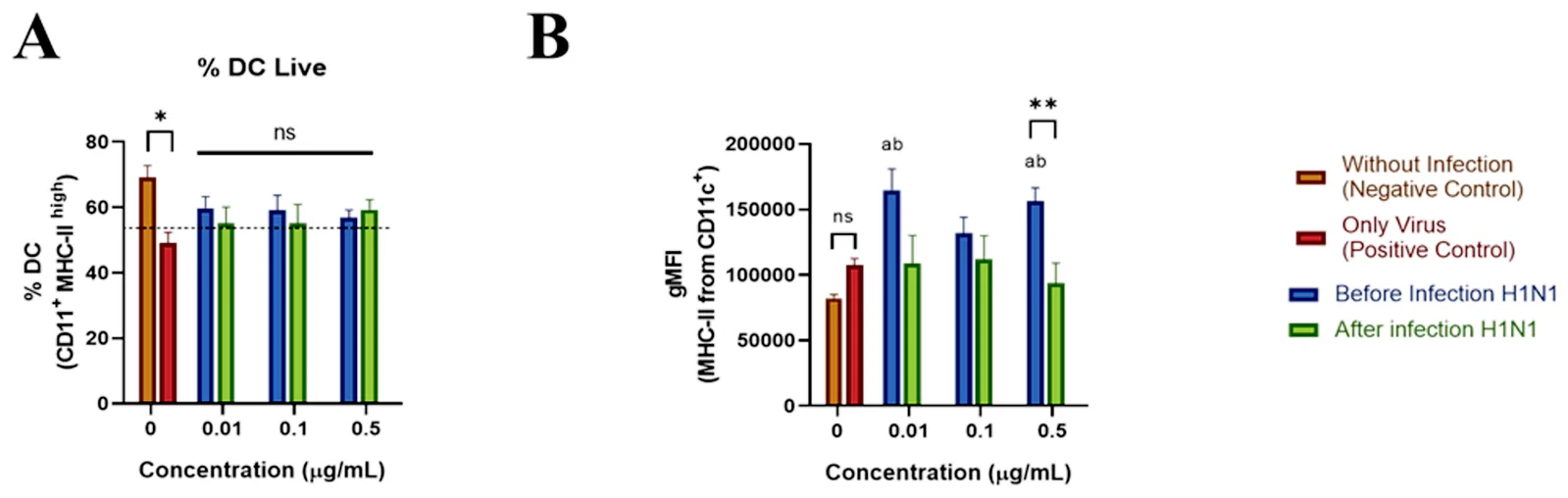
Pulmonarom^®^ increases MHC-II expression after 24 h of H1N1 influenza virus infection. moDCs were either pre-exposed to Pulmonarom^®^ 1 h before infection or treated after the 1-h infection period. **(A)** Percentage of viable CD11c+ HLA-DR+ moDCs recovered. **(B)** Expression of class II major histocompatibility complex (MHC-II) molecules. Data are presented as mean ± standard deviation (SD). Normality was assessed, and comparisons were performed using one-way ANOVA followed by Tukey’s *post hoc* test. (a) statistically different (p < 0.05) compared with the negative control. (b) statistically different (p < 0.05) compared with the positive infection control. Other differences are indicated by *p < 0.05, **p < 0.01. ns, not statistically significant. The dotted line shows the maximum value of the positive control.

**Figure 2:**
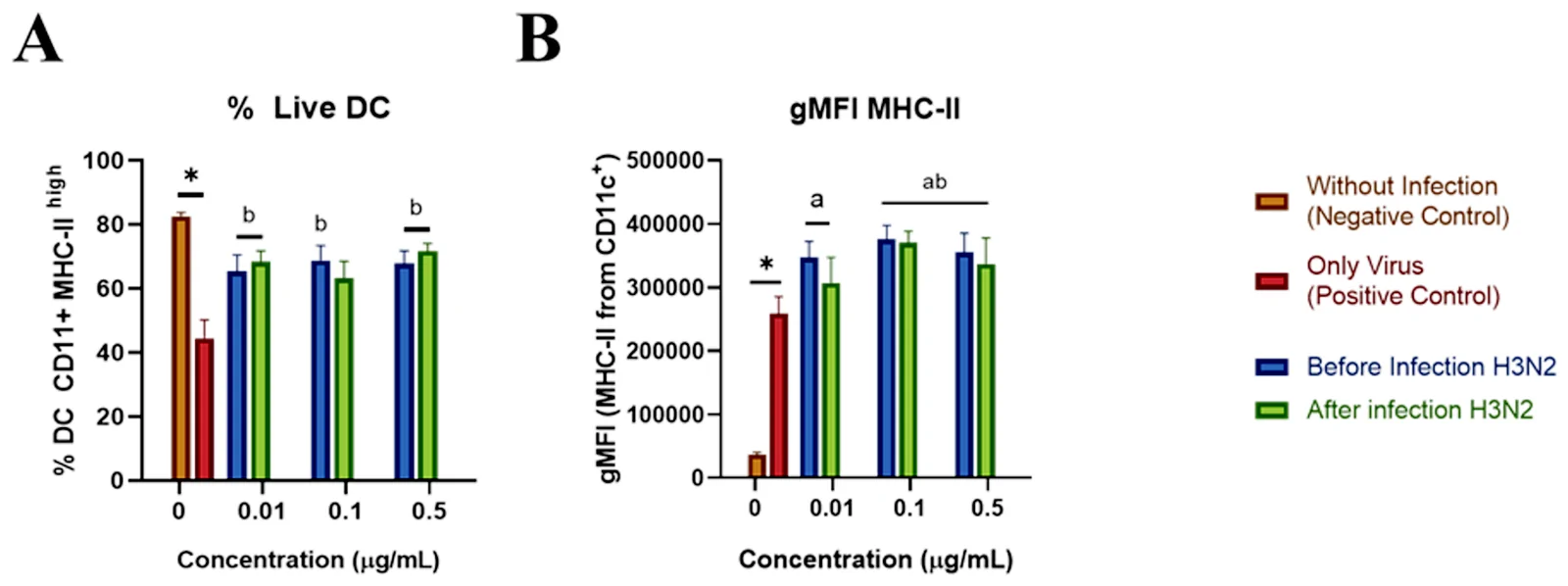
Pulmonarom^®^ increases MHC-II expression after 24 h of H3N2 influenza virus infection. moDCs were either pre-exposed to Pulmonarom^®^ 1 h before infection or treated after the 1-h infection period. **(A)** Percentage of viable CD11c+ HLA-DR+ moDCs recovered. **(B)** Expression of class II major histocompatibility complex (MHC-II) molecules. Data are presented as mean ± standard deviation (SD). Normality was assessed, and comparisons were performed using one-way ANOVA followed by Tukey’s *post hoc* test. (a) statistically different (p < 0.05) compared with the negative control. (b) statistically different (p < 0.05) compared with the positive infection control. Other differences are indicated by *p < 0.05. ns, not statistically significant.

moDCs were either pre-exposed to Pulmonarom^®^ 1 h before infection or treated with Pulmonarom^®^ 1 h after infection with influenza A/H1N1 or A/H3N2 virus. Under both conditions, Pulmonarom^®^ increased MHC class II expression compared with infection alone (**Figures 1**, **2**). Both influenza virus subtypes induced moDC cell death; however, during A/H3N2 virus infection, Pulmonarom^®^ attenuated virus-induced cell death (**Figure 2A**).

**Figure 3:**
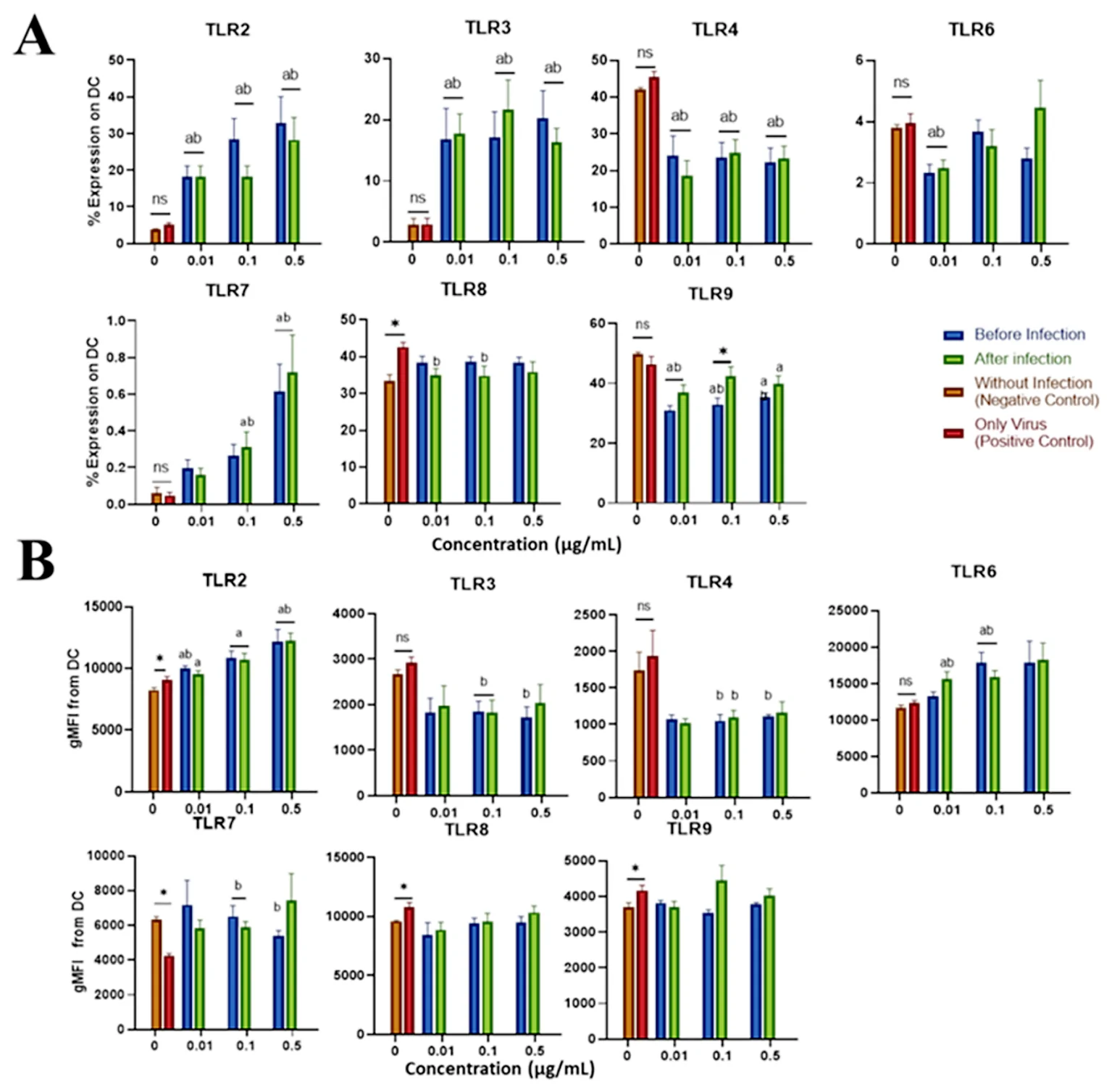
Pulmonarom^®^ increases TLR2, TLR3, TLR6, and TLR7 expression after 24 h of H1N1 influenza virus infection. moDCs were either pre-exposed to Pulmonarom^®^ 1 h before infection or treated after the 1-h infection period. **(A)** Percentage of positive cells and **(B)** geometric mean fluorescence intensity (gMFI) for each TLR. Data are presented as mean ± standard deviation (SD). Normality was assessed, and comparisons were performed using one-way ANOVA with Tukey’s *post hoc* test. (a) statistically different (p < 0.05) compared with the negative control. (b) statistically different (p < 0.05) compared with the positive infection control. Other differences are indicated by *p < 0.05. ns, not statistically significant.

**Figure 4:**
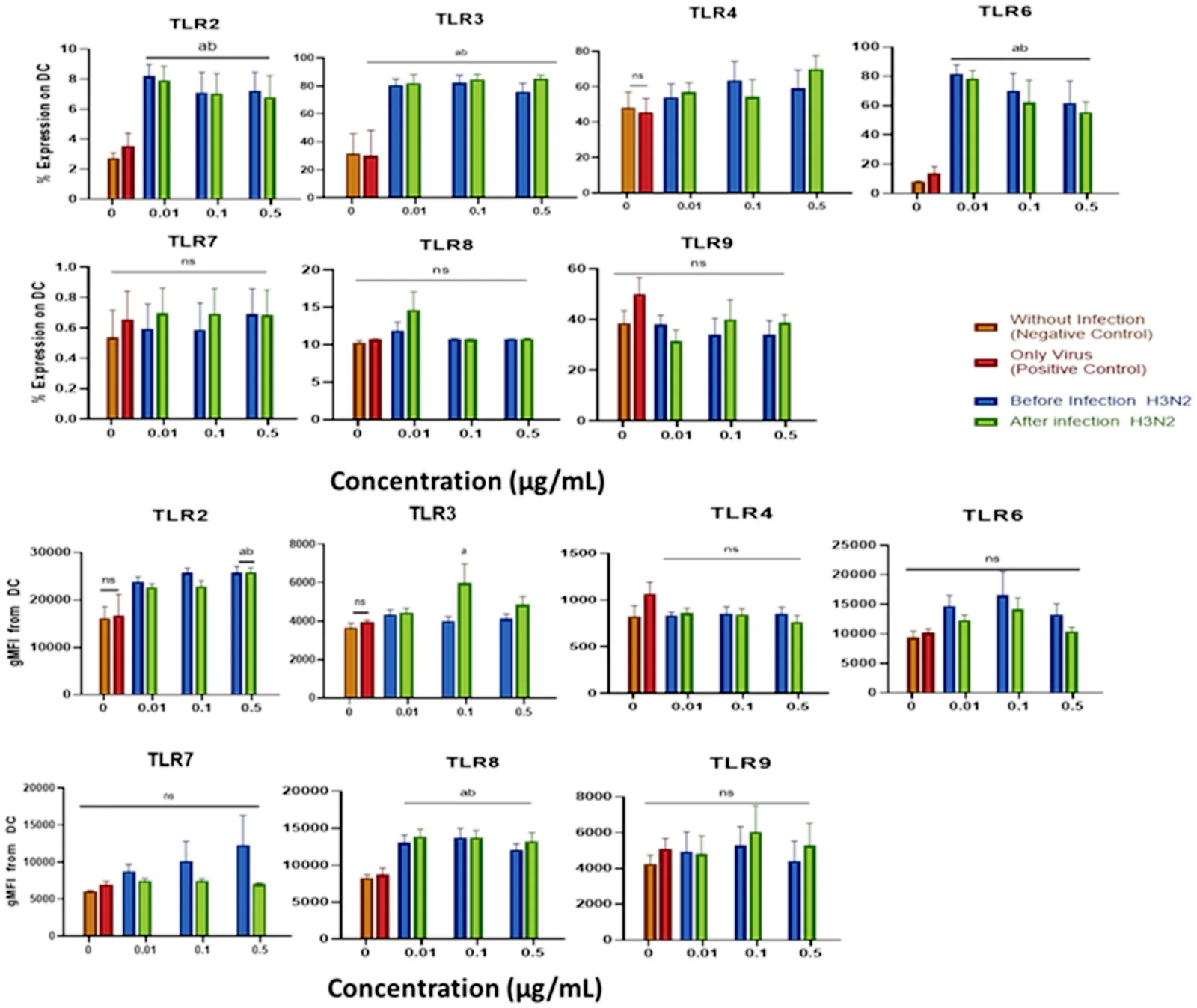
Pulmonarom^®^ increases TLR2, TLR3, TLR6, and TLR8 expression after 24 h of H3N2 influenza virus infection. moDCs were either pre-exposed to Pulmonarom^®^ 1 h before infection or treated after the 1-h infection period. **(A)** Percentage of positive cells and **(B)** geometric mean fluorescence intensity (gMFI) for each TLR. Data are presented as mean ± standard deviation (SD). Normality was assessed, and comparisons were performed using one-way ANOVA followed by Tukey’s *post hoc* test. (a) statistically different (p < 0.05) compared with the negative control. (b) statistically different (p < 0.05) compared with the positive infection control. ns, not statistically significant.

Pulmonarom^®^ also modulated TLR expression in a virus subtype-dependent manner. During A/H1N1 infection, Pulmonarom^®^ increased expression of TLR2, TLR3, TLR6, and TLR7, while decreasing TLR4 expression and the frequency of TLR9-positive cells (**Figure 3**). In contrast, during A/H3N2 infection, Pulmonarom^®^ increased expression of TLR2, TLR3, TLR6, and TLR8 (**Figure 4**).

### 3.2 Pulmonarom^®^ promotes interferon-related antiviral responses

**Figure 5:**
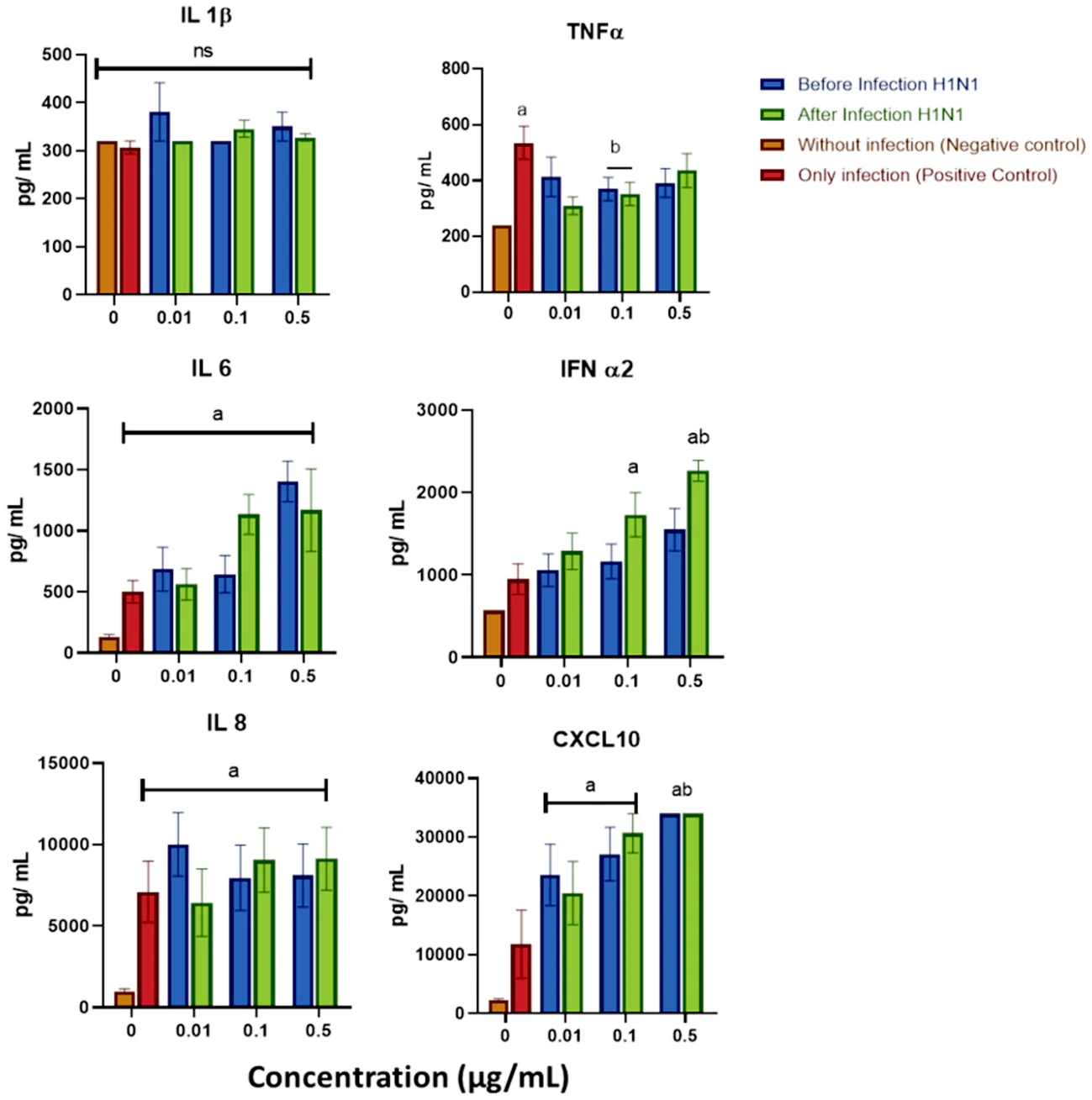
Pulmonarom^®^ increases IFN-α2 and CXCL10 production while reducing TNF-α levels during H1N1 influenza virus infection. Cytokine and interferon production were quantified in supernatants from moDC cultures 24 h after infection and/or Pulmonarom^®^ treatment under the indicated experimental condition. Only cytokines showing statistically significant differences relative to the positive or negative control are presented. Normality was assessed, and comparisons were performed using the Kruskal-Wallis test followed by Dunn’s *post hoc* test. (a) statistically different (p < 0.05) compared with the negative control. (b) statistically different (p < 0.05) compared with the positive infection control. ns, not statistically significant.

**Figure 6:**
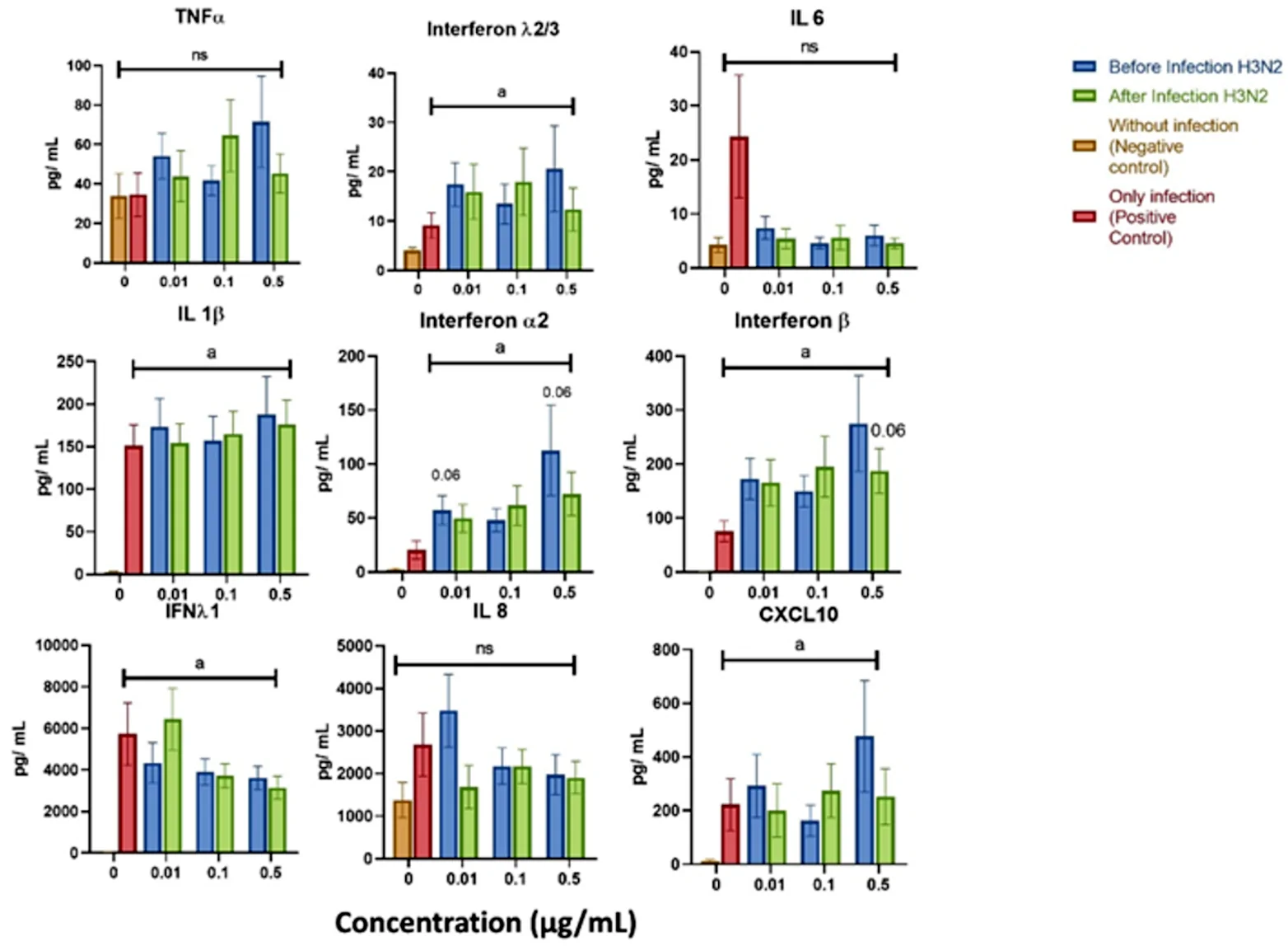
Pulmonarom^®^ shows a trend toward higher IFN-α2 and IFN-β production during H3N2 influenza virus infection. Cytokine and interferon levels were quantified in supernatants from moDC cultures 24 h after infection and/or Pulmonarom^®^ treatment under the indicated experimental condition. Normality was assessed, and comparisons were performed using the Kruskal-Wallis test followed by Dunn’s *post hoc* test. (a) statistically different (p < 0.05) compared with the negative control. (b) statistically different (p < 0.05) compared with the positive infection control. ns, not statistically significant.

Based on these changes in moDC activation, we evaluated whether Pulmonarom^®^ modified cytokine production. During A/H1N1 infection, Pulmonarom^®^ increased IFN-α2 and CXCL10 (also call IP-10) secretion while reducing TNF-α levels (**Figure 5**). During A/H3N2 infection, Pulmonarom^®^ showed a trend toward increased production of type I/III interferon-related cytokines, including IFN-α2, IFN-β, IFN-λ, and CXCL10 (IP-10) (**Figure 6**).

### 3.3 Pulmonarom reduced hemagglutinating activity in viral particles in supernatant of moDCs

**Figure 7:**
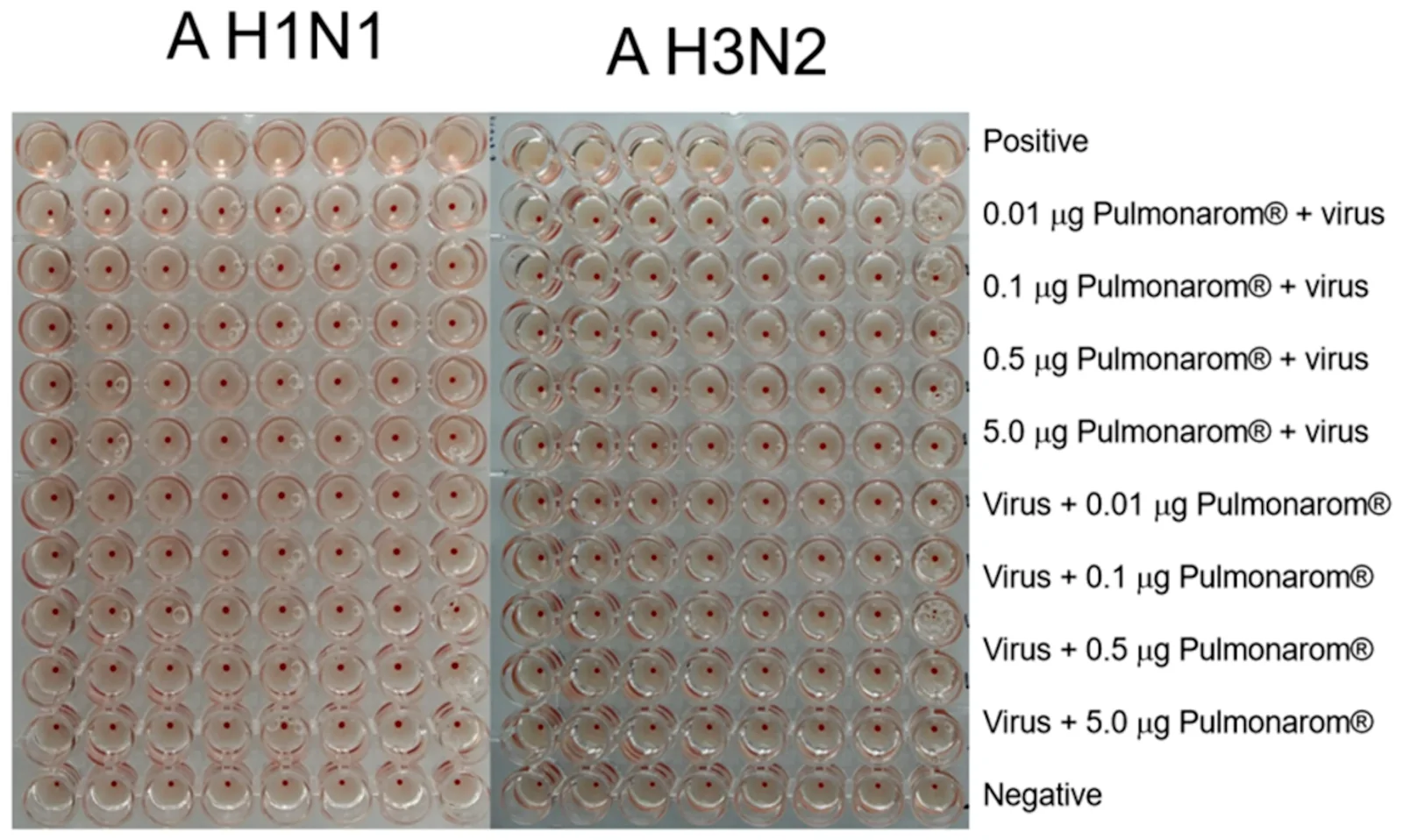
Pulmonarom^®^ reduces hemagglutinating activity induced by influenza A/H1N1 and A/H3N2 virus in culture supernatants. Representative image of the hemagglutination inhibition assay are shown. Supernatants were initially diluted 1:4 and then subjected to two-fold serial dilutions across the plate. In wells with inhibition, erythrocytes formed a compact button at the bottom of the well, whereas in the positive virus control, erythrocytes remained diffusely distributed, consistent with hemagglutination. This assay provides an indirect estimate of hemagglutinating activity and should not be interpreted as a direct measurement of infectious viral titer. (PNR; Pulmonarom).

Although influenza virus infection alone induced cytokine production, Pulmonarom^®^ further increased selected cytokines under both pre-exposure and post-infection treatment conditions. In parallel, Pulmonarom^®^ treatment was associated with reduced hemagglutinating activity in moDCs culture supernatants (**Figure 7**, **Table 1**). Because hemagglutination-based assays do not directly quantify infectious viral progeny, these data should not be interpreted as infectious virus titers.

**Table 1:** Hemagglutination inhibition titers for influenza A/H1N1 and A/H3N2 virus under the indicated experimental conditions.

Condition 1	Condition 2	Inhibition of hemaglutination	Title	Result	Condition 1	Condition 2	Inhibition of hemaglutination	Title	Result
H1N1	0.01 μg PNR	NO	0	0	H3N2	0.01 μg PNR	NO	0	0
H1N1	0.01 μg PNR	NO	0		H3N2	0.01 μg PNR	NO	0	
H1N1	0.01 μg PNR	NO	0		H3N2	0.01 μg PNR	NO	0	
0.01 μg PNR	H1N1	NO	0	0	0.01 μg PNR	H3N2	NO	0	0
0.01 μg PNR	H1N1	NO	0		0.01 μg PNR	H3N2	NO	0	
0.01 μg PNR	H1N1	NO	0		0.01 μg PNR	H3N2	NO	0	
H1N1	0.1 µg PNR	NO	0	0	H3N2	0.1 μg PNR	NO	0	0
H1N1	0.1 µg PNR	NO	0		H3N2	0.1 µg PNR	NO	0	
H1N1	0.1 μg PNR	NO	0		H3N2	0.1 μg PNR	NO	0	
0.1 µg PNR	H1N1	NO	0	0	0.1 μg PNR	H3N2	NO	0	0
0.1 µg PNR	H1N1	NO	0		0.1 μg PNR	H3N2	NO	0	
0.1 μg PNR	H1N1	NO	0		0.1 ug PNR	H3N2	NO	0	
H1N1	0.5 µg PNR	NO	0	0	H3N2	0.5 μg PNR	NO	0	0
H1N1	0.5 μg PNR	NO	0		H3N2	0.5 μg PNR	NO	0	
H1N1	0.5 μg PNR	NO	0		H3N2	0.5 μg PNR	NO	0	
0.5 µg PNR	H1N1	NO	0	1:2	0.5 μg PNR	H3N2	NO	0	0
0.5 µg PNR	H1N1	NO	2		0.5 μg PNR	H3N2	NO	0	
0.5 μg PNR	H1N1	NO	0		0.5 μg PNR	H3N2	NO	0	
H1N1	5 µg PNR	NO	0	0	H3N2	5 μg PNR	NO	0	0
H1N1	5 µg PNR	NO	0		H3N2	5 Hg PNR	NO	0	
H1N1	5 µg PNR	NO	0		H3N2	5 Hg PNR	NO	0	
5 µg PNR	H1N1	NO	0	0	5 µg PNR	H3N2	NO	0	0
5 µg PNR	H1N1	NO	0		5 µg PNR	H3N2	NO	0	
5 μg PNR	H1N1	NO	0		5 ug PNR	H3N2	NO	0	
H1N1	NA	YES	32	1:32	H3N2	NA	YES	256	1:256
H1N1	NA	YES	32		H3N2	NA	YES	256	
H1N1	NA	YES	32		H3N2	NA	YES	256	
NA	NA	NO	0	0	NA	NA	NO	0	0
NA	NA	NO	0		NA	NA	NO	0	
NA	NA	NO	0		NA	NA	NO	0	

Titers were determined from two-fold serial dilutions starting at 1:4 and measured in triplicate. Values represent inhibition of hemagglutinating activity rather than infectious viral titers.

### 3.4 Reduction of plaque-forming viral particles in moDC supernatants by Pulmonarom^®^

**Figure 8:**
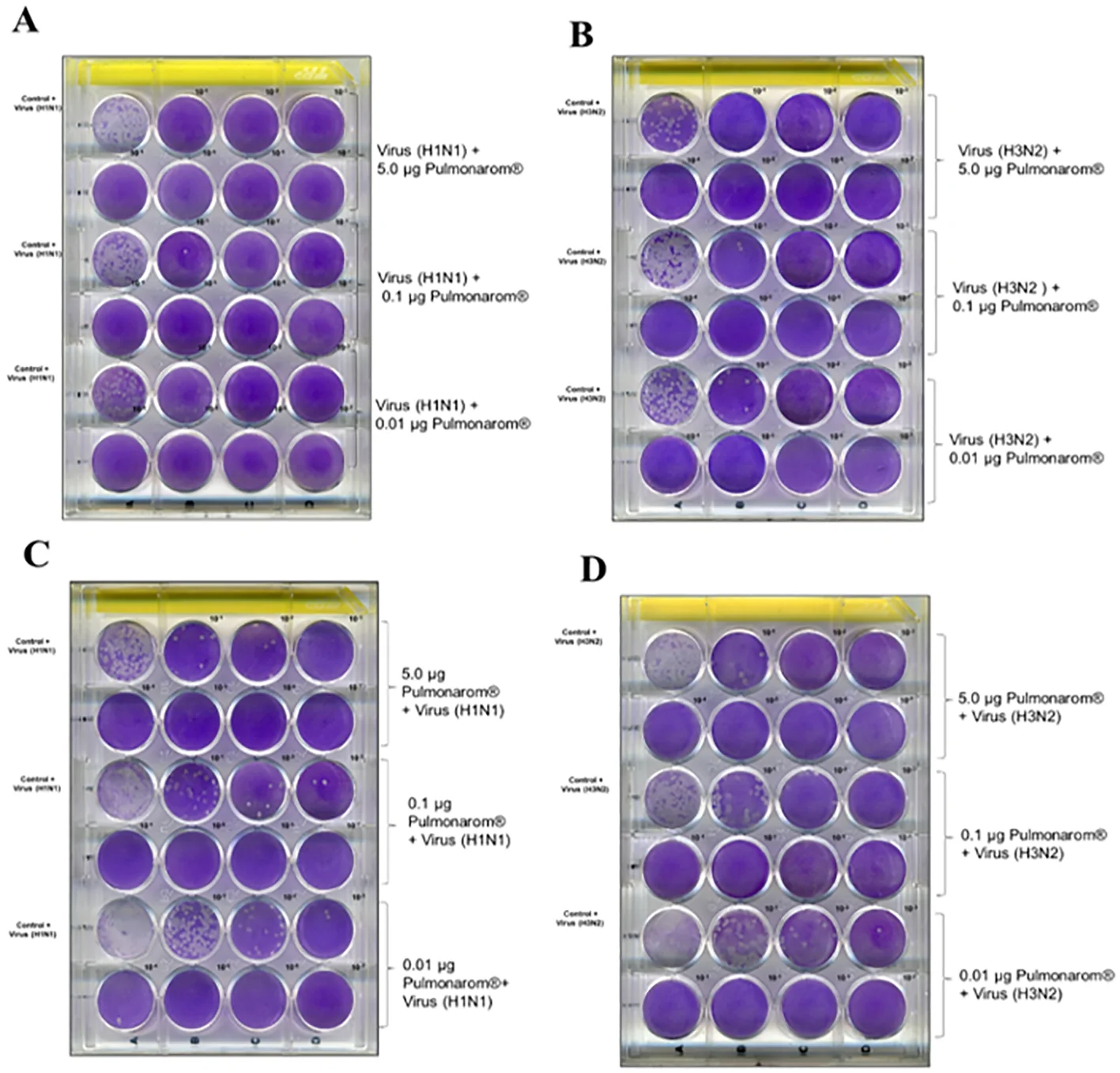
Representative images of plaque assays evaluating the supernatants from moDCs infected with influenza A virus. Both post-treatment **(A, B)** and pre-treatment **(C, D)** conditions were assessed, using influenza A/H1N1 **(A, C)** and A/H3N2 **(B, D)**. A significant reduction in the number of cell-lysis plaques was observed, demonstrating the antiviral effect of Pulmonarom^®^ treatment, with the post-infection condition showing the strongest effect. The experiment was performed in triplicate, consistently yielding similar results across independent experiments.

The antiviral activity of Pulmonarom^®^ against influenza A/H1N1 and A/H3N2 viruses was evaluated by plaque assay using supernatants collected from moDCs treated with Pulmonarom^®^ either before (pretreatment) or after (post-treatment) viral infection. In the post-treatment setting, Pulmonarom^®^ inhibited viral replication in a concentration-dependent manner against both influenza A subtypes (H1N1 and H3N2). In moDCs infected with influenza A/H1N1, treatment with 5.0 µg of Pulmonarom^®^ completely abolished detectable infectious virus, as no plaques were observed. In contrast, treatment with 0.1 µg and 0.01 µg reduced viral titers to 1 × 10² PFU/mL and 5 × 10² PFU/mL, respectively (**Figure 8A**). A similar antiviral effect was observed against influenza A/H3N2. Treatment with 5.0 µg completely suppressed the production of infectious viral particles, whereas treatment with 0.1 µg and 0.01 µg reduced viral titers to 2 × 10² PFU/mL and 7 × 10² PFU/mL, respectively (**Figure 8B**).

Pulmonarom^®^ also reduced viral replication in the pretreatment setting in a concentration-dependent manner, although the antiviral effect was less pronounced than that observed with post-treatment. In moDCs pretreated with Pulmonarom^®^ prior to influenza A/H1N1 infection, treatment with 5.0 µg resulted in a viral titer of 4 × 10³ PFU/mL, whereas treatment with 0.1 µg and 0.01 µg yielded titers of 2 × 10^4^ PFU/mL and 1 × 10^5^ PFU/mL, respectively (**Figure 8C**). Likewise, for influenza A/H3N2, treatment with 5.0 µg reduced the viral titer to 5 × 10² PFU/mL, while treatment with 0.1 µg and 0.01 µg resulted in titers of 5 × 10³ PFU/mL and 1 × 10^4^ PFU/mL, respectively (**Figure 8D**). Overall, both treatment regimens demonstrated significant antiviral activity against influenza A/H1N1 and A/H3N2 viruses. The inhibitory effect was concentration-dependent and was more pronounced when Pulmonarom^®^ was administered after viral infection. In both experimental settings, viral titers were markedly lower than those observed in the infected control, which reached approximately 1 × 10^8^ PFU/mL for both influenza A subtypes (**Figure 8**).

### 3.5 Pulmonarom^®^ is associated with ultrastructural changes in H3N2-infected moDCs

**Figure 9:**
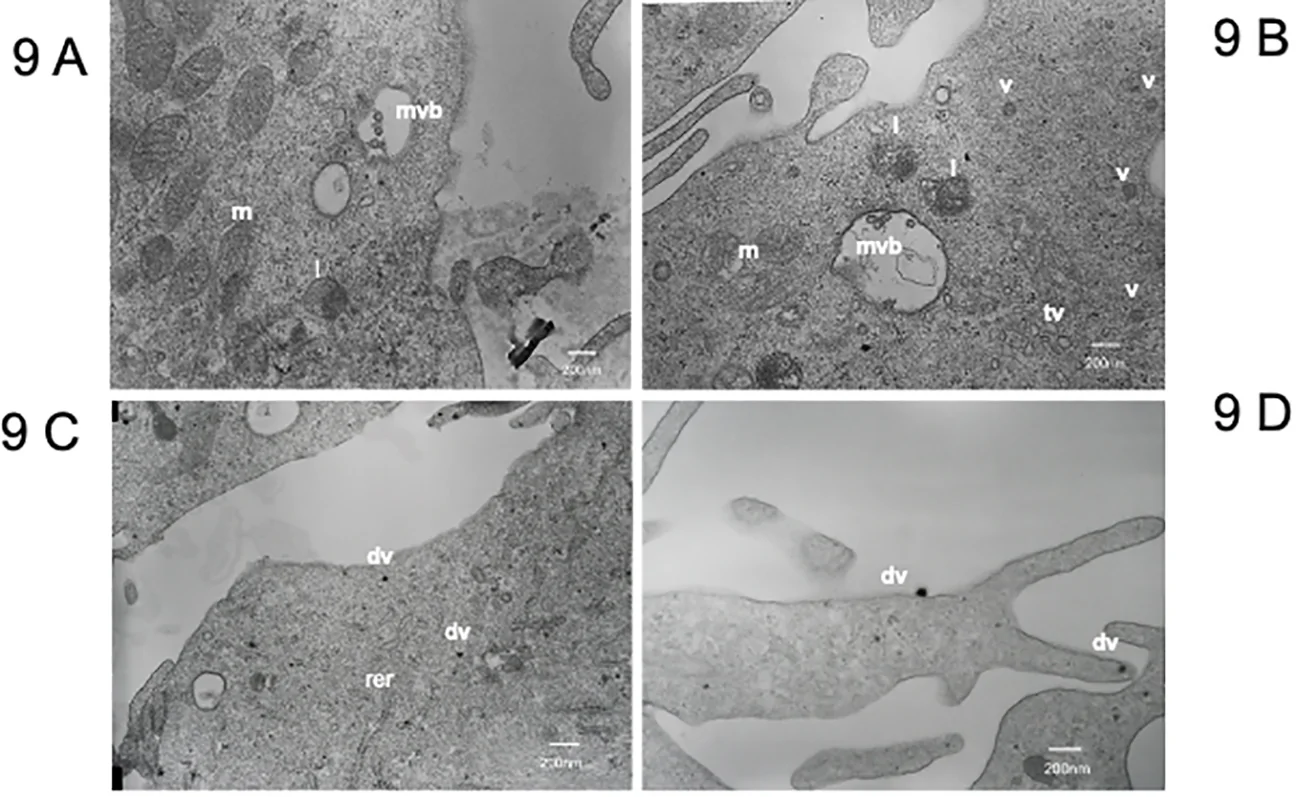
Transmission electron microscopy (TEM) of moDCs after H3N2 influenza virus infection **(A)** Negative control. **(B)** Positive infection control, moDCs infected with influenza A/H3N2 virus. **(C)** Pre-exposure condition, in which moDCs were exposed to Pulmonarom^®^ before influenza A/H3N2 virus infection. **(D)** Post-infection treatment condition, in which Pulmonarom^®^ was added after the 1-h infection period. m, mitochondria; l, lysosome; v, virus-like particle; mvb, multivesicular body; tv, transport vesicles; rer, rough endoplasmic reticulum; avp, altered virus-like particles. Because no virus-specific labeling was performed, identification of virus-like structures is based on morphology. TEM analysis was performed for H3N2 only.

Transmission electron microscopy provided exploratory ultrastructural information for A/H3N2-infected moDCs (**Figure 9**). Uninfected, unstimulated moDCs in the negative control group (**Figure 9A**) showed preserved cellular ultrastructure, including ribosomes, elliptical mitochondria with organized cristae and electron-dense matrix, transport vesicles, and multivesicular bodies.

In moDCs infected with A/H3N2 virus (positive infection control, **Figure 9B**), mitochondrial collapse and loss of cristae organization were observed. In the vacuolated cytoplasm, small, electron-dense circular structures of approximately 100 nm were detected. These structures were compatible in size and morphology with influenza virus-like particles.

In the pre-exposure group (**Figure 9C**), moDCs exposed to Pulmonarom^®^ before A/H3N2 virus infection showed better preservation of mitochondrial morphology than infected controls. Electron-dense cytoplasmic structures with irregular morphology and variable size were also observed. These features may correspond to altered virus-like particles or intracellular vesicular structures; however, without specific viral labeling, they cannot be classified as defective viral particles.

In the post-infection treatment group (**Figure 9D**), moDCs were first infected and then treated with Pulmonarom^®^. These cells showed marked vacuolization and cytopathic changes, together with mitochondrial alterations near large vacuolar structures. Bundles suggestive of cytoskeletal filaments were also observed. These findings indicate that Pulmonarom^®^ treatment is associated with ultrastructural changes during H3N2 infection.

Taken together, the TEM observations should be considered descriptive and exploratory. They suggest changes in viral particle morphology or vesicular processing in Pulmonarom^®^-treated H3N2-infected cells, but they do not demonstrate defective infectious progeny.

## 4 Discussion

In our previous study, Pulmonarom^®^ activated human monocyte-derived dendritic cells (moDCs), as shown by increased MHC class II expression, greater lysosomal activity, and upregulation of selected TLRs and cytokines (Hernández-Aceves et al., 2025) The present study extends those observations to an *in vitro* influenza A virus infection model and shows that Pulmonarom^®^ modifies moDC responses under both pre-exposure and post-infection treatment conditions.

Pulmonarom^®^ increased MHC-II expression during infection with both influenza A/H1N1 and A/H3N2 virus (**Figures 1**, **2**). This finding supports an antigen-presentation-related activation profile during viral challenge. However, because additional activation and maturation markers, such as CD80, CD86, CD83, CD40, among others, as well as influenza-specific T-cell activation, were not evaluated, the data should not be interpreted as evidence of full dendritic-cell maturation or increased antigen-specific T-cell priming. The terms pre-exposure and post-infection treatment are used only to describe the timing of Pulmonarom^®^ exposure *in vitro* and do not imply clinical preventive or therapeutic efficacy.

Pulmonarom^®^ also attenuated virus-induced cell death during A/H3N2 infection (**Figure 2A**), suggesting that the lysate may help preserve moDC viability under viral stress. Although the underlying mechanism was not examined, maintenance of moDC viability could contribute to the preservation of innate immune sensing, cytokine production, and antigen-presentation-related functions during infection. Whether this effect involves modulation of cell-death pathways, cellular metabolism, or viral replication will require further investigation.

A relevant finding was the subtype-dependent pattern of TLR expression. During A/H1N1 infection, Pulmonarom^®^ increased TLR2, TLR3, TLR6, and TLR7, while reducing TLR4 expression and the frequency of TLR9-positive cells (**Figure 3**). During A/H3N2 infection, it increased TLR2, TLR3, TLR6, and TLR8 (**Figure 4**). This pattern is biologically plausible because TLRs participate in microbial and viral sensing, interferon induction, and antiviral responses (Liu et al., 2012; Dowling and Mansell, 2016; Zhou et al., 2021). Nevertheless, these results should be interpreted as TLR-related phenotypic changes rather than evidence of direct receptor dependency, since receptor-blocking or signaling-inhibition experiments were not performed.

The different TLR7- and TLR8-related profiles observed during A/H1N1 and A/H3N2 infection may reflect differences in viral replication kinetics, viral RNA availability, endosomal processing, cellular stress, or infection efficiency. Both virus subtypes were tested at the same nominal multiplicity of infection and evaluated at the same 24-h post-infection time point; however, equivalent infectious input and intracellular viral load were not independently confirmed by viral RNA quantification or viral-protein staining. Therefore, the apparent subtype-specific differences should be interpreted cautiously and validated through kinetic and mechanistic studies (Frensing et al., 2016; Rüdiger et al., 2019).

Cytokine analysis further supports an antiviral immunomodulatory effect. During A/H1N1 virus infection, Pulmonarom^®^ increased IFN-α2 and CXCL10 while reducing TNF-α, suggesting preservation of interferon-related antiviral responses with more limited inflammatory signaling (**Figure 5**). This interpretation should remain cautious because TNF-α can contribute to antiviral defense during influenza infection, but it can also participate in inflammatory tissue injury and immunopathology depending on the context and timing of infection (Jewell et al., 2010; Lozhkov et al., 2020). Thus, the reduction in TNF-α at 24 h should be considered a time-specific observation rather than evidence that TNF-α is dispensable.

During A/H3N2 virus infection, Pulmonarom^®^ showed trends toward increased IFN-α2, IFN-β, IFN-λ, and CXCL10 (**Figure 6**). Although some changes were modest, the overall profile is consistent with an increase in interferon-related responses. IFN-λ is particularly relevant in respiratory antiviral defense, including influenza virus infection (Jewell et al., 2010; Lozhkov et al., 2020). Because cytokines were evaluated at a single 24-h post-infection time point, future kinetic analyses should include earlier and later intervals to distinguish transient inflammatory responses from sustained antiviral activity.

The immunological changes induced by Pulmonarom^®^ were accompanied by reduced hemagglutinating activity in culture supernatants. This association is compatible with the possibility that Pulmonarom^®^ reinforces innate antiviral defenses in moDCs. However, hemagglutination-based assays do not directly measure infectious viral progeny because noninfectious or defective particles may retain hemagglutinin-mediated red blood cell agglutinating activity. Therefore, these results should be interpreted as reduced hemagglutinating activity rather than direct evidence of reduced infectivity. Future studies should include plaque assay or titration, as well as cell-free virus-Pulmonarom^®^ incubation assays, to determine whether Pulmonarom^®^ directly affects influenza virions independently of immune-cell activation (Frensing et al., 2016; Rüdiger et al., 2019).

Our findings agree with previous reports showing that bacterial lysates can modulate respiratory immune responses, although their effects are likely product-specific. Roth et al. reported distinct antiviral properties of different bacterial lysates, including Pulmonarom^®^ and OM-85, in respiratory virus models (Roth et al., 2021). In addition, previous Pulmonarom^®^-related studies support activity in human monocyte/macrophage models, including BPI induction, autophagy-associated antimicrobial responses, and innate signatures favorable to macrophage responses in tuberculosis infection (García-González et al., 2025; Romero-Rodríguez et al., 2025). Because this study employed moDCs rather than freshly isolated primary monocytes as the final experimental population, important questions regarding monocyte viability, activation marker expression, and inflammatory cytokine production during influenza virus infection remain unresolved and represent a critical avenue for future investigation.

Other dendritic-cell-targeting immunomodulators provide useful context for interpreting the present findings. Pidotimod is a synthetic dipeptide traditionally used to prevent recurrent respiratory infections. Experimental studies have shown that it promotes functional maturation of human dendritic cells, increasing HLA-DR, CD83, and CD86 expression, stimulating cytokine and chemokine production, and supporting T-cell proliferation and Th1-oriented responses (Giagulli et al., 2009; Ucciferri et al., 2021; Marseglia et al., 2025; Ucciferri et al., 2025). More recent clinical reviews have proposed extending its potential applications beyond the prevention of recurrent infections to include complementary use in pneumonia, chronic respiratory diseases, and selected viral infections (Marseglia et al., 2025; Ucciferri et al., 2025). A pilot study in virologically suppressed individuals with HIV also reported cytokine rebalancing and increased salivary IgA after pidotimod supplementation, suggesting broader systemic immunomodulatory activity, although dendritic-cell responses were not directly evaluated in that clinical setting (Ucciferri et al., 2021). Pidotimod has been shown experimentally to increase HLA-DR, CD83, and CD86 and to favor Th1-oriented responses, whereas the HIV study mainly supports broader immune rebalancing rather than a specifically demonstrated DC-mediated mechanism.

Receptor-selective compounds provide another comparison. Monophosphoryl lipid A acts primarily as a TLR4 agonist with TRIF-biased signaling and is principally used as a vaccine adjuvant (Mata-Haro et al., 2007). In contrast, multicomponent bacterial lysates contain multiple microbial-associated molecular patterns that may simultaneously engage several pattern-recognition pathways. OM-85 represents a particularly relevant comparator because, like Pulmonarom^®^, it is a multibacterial lysate used in respiratory infection prevention. In human dendritic cells, OM-85 was reported to activate NF-κB- and MAPK-dependent pathways and induce selected chemokines and B-cell-supporting cytokines, with no detectable activation of the IRF pathway under the experimental conditions examined (Parola et al., 2013).

Although direct comparisons among studies are limited by differences in formulation, concentration, experimental system, and infection status, Pulmonarom^®^ showed potentially distinguishing features in the present model. These included modulation of several TLRs, increased MHC-II expression, interferon- and CXCL10-related responses, preservation of moDC viability during A/H3N2 infection, and reduced recoverable infectious virus. Its multicomponent composition may allow broader and context-dependent engagement of innate immune pathways than single-molecule or receptor-selective immunomodulators. This should not be interpreted as evidence of superiority, which would require standardized head-to-head comparisons.

An additional point of interest is that Pulmonarom^®^ showed activity under both pre-exposure and post-infection treatment conditions. Bacterial lysates and pidotimod have traditionally been considered mainly for prevention of recurrent respiratory infections, although recent literature has increasingly discussed their use as complementary interventions during established infections. The present post-infection findings therefore broaden the experimental rationale for Pulmonarom^®^, suggesting that its potential applications may not be restricted to prophylactic immune stimulation but could also include host-directed support after viral infection has begun. The recent pidotimod literature similarly discusses a transition from conventional prevention toward broader complementary applications in respiratory and viral disease. Nevertheless, this interpretation remains hypothesis-generating because the present results were obtained *in vitro* and do not establish treatment efficacy, optimal timing, or clinical benefit.

The TLR-related changes and interferon responses observed here, together with previous reports in monocyte/macrophage models, are compatible with innate immune priming. However, trained immunity requires evidence of long-term functional reprogramming, metabolic or epigenetic remodeling, and increased secondary responses after restimulation (Hu et al., 2022; Dagenais et al., 2023; Hu et al., 2026). These features were not evaluated here; therefore, Pulmonarom^®^-associated changes should be described as activation-related or compatible with priming rather than as definitive evidence of trained immunity.

Hemagglutination inhibition and plaque titration assays performed with supernatants from influenza-infected moDCs demonstrated that Pulmonarom^®^ can reduce the infective capacity of residual viral particles in the supernatant. However, this result should be interpreted with caution, as it does not necessarily imply that the same outcome would occur in *in vivo* systems; further validation through viral challenge experiments is required (**Figures 7**, **8**).

Transmission electron microscopy provided complementary but only exploratory structural information. Virus-like structures were observed in A/H3N2-infected moDCs, and Pulmonarom^®^ exposure was associated with altered ultrastructural features and partial preservation of mitochondrial morphology (**Figure 9**). However, these observations were based on conventional morphology and were not confirmed by specific viral labeling. Therefore, the observed structures should be regarded as virus-like or morphologically suggestive, and autophagy-related interpretations require additional validation (Eskelinen, 2008; Hidalgo et al., 2016; Wolff and Bárcena, 2021; Lara-Hernandez et al., 2023; Stelitano and Cortese, 2024). Parallel TEM analysis of A/H1N1- and A/H3N2-infected cultures will also be needed to determine whether the ultrastructural changes are subtype-specific.

Overall, Pulmonarom^®^ induced an antiviral activation profile in human moDCs during influenza A virus infection, involving increased MHC-II expression, TLR-related phenotypic changes, stronger interferon/CXCL10 responses, and reduced hemagglutinating activity. These findings support the further study of Pulmonarom^®^ as an immunomodulatory strategy targeting innate immune pathways relevant to antiviral defense. At the same time, the study should be interpreted within its experimental scope.

Several limitations should be acknowledged. This study was conducted *in vitro* using moDCs, which do not fully recapitulate the complexity of the respiratory immune environment. Mechanistic pathways were not directly dissected, and cytokine analyses were limited to predefined time points. In addition, adaptive immune consequences were not evaluated.

Future studies should incorporate Endotoxin quantification, receptor-blocking experiments, infection kinetics, direct quantification of infectious viral progeny, expanded maturation-marker panels, T-cell functional validation, and *in vivo* studies will be required to define the pathways involved and assess translational relevance.

## 5 Conclusion

This study indicates that Pulmonarom^®^ can modify human moDC responses during influenza A virus infection, favoring an innate antiviral profile under both pre-exposure and post-infection treatment conditions. These findings support further investigation of bacterial lysates as host-directed immunomodulatory strategies for respiratory viral infections. Additional mechanistic, functional, and *in vivo* studies are needed to determine whether this moDC activation profile translates into improved antiviral protection and adaptive immune priming.

## Data Availability

The raw data supporting the conclusions of this article will be made available by the authors, without undue reservation.
